# ENETS TNM Staging Predicts Prognosis in Small Bowel Neuroendocrine Tumours

**DOI:** 10.1155/2013/420795

**Published:** 2013-02-28

**Authors:** Rajaventhan Srirajaskanthan, A. Ahmed, A. Prachialias, P. Srinivasan, N. Heaton, N. Jervis, A. Quaglia, G. Vivian, J. K. Ramage

**Affiliations:** ^1^Institute of Liver Studies, King's College Hospital, London SE5 9RS, UK; ^2^Department of Gastroenterology, University Hospital Lewisham, London SE13 6LH, UK; ^3^Nuclear Medicine, King's College Hospital, London SE5 9RS, UK; ^4^Department of Gastroenterology, Basingstoke and North Hampshire Foundation Trust, Hampshire R924 9NA, UK

## Abstract

*Introduction*. Small bowel neuroendocrine tumours (NETs) are the most common type of gastrointestinal neuroendocrine tumours. The incidence and prevalence of these tumours are on the rise. The aims of this study were to determine prognostic clinicopathological features and whether the ENETS TNM staging system predicts prognosis and also. *Method*. Clinical data was collected retrospectively from 138 patients with histologically proven small bowel NETs managed at King's College Hospital. Histology was reviewed and small bowels tumours, were staged according to the ENETS TNM staging system. *Results*. Median age was 65 years (range 29–87). The 5-year survival was 79.5% and the 10-year survival was 48.5%. Resection of the primary tumour was associated with improved survival (120 versus 56 months, *P* < 0.05). On multivariate analysis prognostic factors were primary tumour resection and not having a carcinoid heart disease. TNM staging significantly separated survival of stage 2 and stage 3 from stage 4 NETs. *Conclusion*. Small bowel primary tumour resection and not having carcinoid heart disease are prognostic factors. The ENETS TNM staging and grading system appears to be of prognostic relevance to small bowel NETs.

## 1. Introduction

Neuroendocrine tumours of the small bowel are the most common type of malignant neoplasm in the small intestine, accounting for 35% of small intestinal cancers [1, 2]. Small bowel neuroendocrine tumours (NETs) are the most common type of gastrointestinal neuroendocrine tumours [3]. Small bowel NETs comprise around 38% of gastroenteropancreatic NETs and 21% of all NETs. The incidence and prevalence of these tumours are on the rise, as demonstrated in the Surveillance Epidemiology and End Results (SEER) data and the population-based study in Norway [2, 4]. A threefold increase in incidence has been demonstrated in the USA between 1973 and 2002 [5]. The reported incidence of small bowel NETs is 1/100 000 population [6]. The majority of these tumours do not cause carcinoid syndrome, often presenting late with metastatic disease. Patients with non-hormone secreting tumours often present with vague symptoms, including intermittent abdominal pain or weight loss. A number of patients are identified coincidentally. Approximately 40% of patients with metastatic disease at presentation have functionally active tumours leading to the development of carcinoid syndrome [7–10]. 

Small bowel NETs were generally thought to be indolent tumours; however, their behaviour is more heterogeneous, and consequently a staging and grading system has been introduced by European Neuroendocrine Tumour Society (ENETS) to help clinicians to optimize the management of these patients [11]. The ENETS grading system incorporates Ki67 index and mitotic rate to grade tumours; these parameters had not previously been incorporated to the WHO 2000 classification of NETs. 

Yao et al. demonstrated a median survival from presentation of 65 months for patients with stage 4 (distant metastatic) well to moderately differentiated small bowel NETs [6]. More recent studies have demonstrated 5-year survival of >70% for patients with metastatic small bowel NETs [12, 13]. 

A number of studies have assessed the survival benefit of primary tumour resection and other therapeutic interventions [12, 14–17]. However, there is still no consensus as to whether primary resection in patients with distal metastatic disease at presentation is beneficial. There is conflicting evidence regarding the survival benefit of liver resection, though it is generally recommended to offer liver resection if complete tumour removal or debulking of >90% of the liver disease is possible [18]. 

To date there is limited data regarding the prognostic relevance of the proposed ENETs TNM staging and grading system [13], which has been validated in foregut and a combined study of mid-and hindgut NETs [13, 19]. In this study we report our experience with small bowel NETs over a 20-year period (from 1990 to 2010). The aims of this study were twofold: firstly to determine whether the ENETS TNM staging system predicts prognosis in patients with small bowel NETs and secondly, to determine prognostic clinicopathological factors in patients with small bowel NETs. 

## 2. Materials and Methods

Patients with small bowel primary NETs were identified through a search on the neuroendocrine tumour database at King's College Hospital, London, UK. The medical records of 181 patients were analysed. Patients with tumours arising from the ampulla of Vater and ileocaecal valve were excluded from analysis. Patients with unknown primary were not included. Site of primary and assessment of metastatic disease were based on operative, cross-sectional, and/or nuclear medicine imaging. Data collection was performed by two investigators (R. Srirajaskanthan and A. Ahmed) using a specifically designed database (Filemaker Pro). Forty-three patients were excluded from analysis due to incomplete clinical records. 

One hundred and thirty-eight patients were included in the study. The date of diagnosis was from 1990 to 2010. All patients had histological confirmation based on surgical specimen or liver biopsy. In all cases histological diagnosis was based on the microscopy and when possible immunohistochemical staining with NET markers [20]. A second histological assessment was performed at King's College Hospital when possible to confirm accurate histological characterisation and grading of tumours.

The TNM staging system proposed by ENETS was used to stage patients in whom complete histological and radiological assessment was possible [11, 21]. Radiological assessment included a CT of chest, abdomen, and pelvis plus an octreotide scan in all cases. MIBG scans were performed in some instances. Due to the small number of patients with stage 2a disease and stage 2b disease, these were amalgamated together for analysis. Stage 3a and stage 3b disease was also analysed as one group. 

 The Histological assessment for tumour grade using the proposed ENETS classification was performed in all cases where histology was available. Grade 1 is classified as a Ki67 ≤2%, Grade 2 Ki67 3–20%, and Grade 3 Ki67 >20% [11, 21]. Study population demographics are displayed in Table 1. 

Patients underwent a number of therapeutic interventions in this study including surgery and biotherapy with somatostatin analogues. Peptide receptor targeted therapy in this study comprised of both ^90^Yttrium-DOTATATE therapy and since 2008 ^177^Lu-DOTATATE therapy. Selective internal radiation therapy (SIRT) was performed on carefully selected individuals; a treatment was regarded as embolization of one lobe of the liver. Therefore, if patients had both lobes treated, this would be regarded as two treatments. Radiofrequency ablation (RFA) was performed either percutaneously or surgically.  Table 2 illustrates the number of different therapeutic interventions patients underwent during the course of their treatment. 

### 2.1. Statistical Analysis

Continuous variables are reported as mean ± SD or median (range) if not normally distributed. Survival was measured from the time of diagnosis to death. Survival curves were constructed using Kaplan Meier method for analysis of censored data. Log rank tests were performed to compare survival between groups. Throughout all analyses, statistical significance was determined by a criterion of *P* < 0.05. Multivariate analysis of predictors of death was by Cox regression. Histological grade was assessed in univariate analysis but was excluded from multivariate analysis due to the amount of censored data leading to insufficient numbers to perform multivariate analysis. Variables were removed stepwise from the model when *P* value exceeded 0.10 and variables with a *P* < 0.05 in the final model were considered significant predictors of death. Data analysis was performed using GraphPad Prism software (GraphPad Prism version 5.00 for Windows, GraphPad Software, San Diego California USA, http://www.graphpad.com/) and SPSS V.16 (SPSS Inc.).

## 3. Results

### 3.1. Clinical and Tumour Characteristics

The median age of the 138 patients was 65 years (range 29–87) at time of diagnosis; there were 68 males and 70 females. The 76 patients (55%) had functional tumours, and the remainder were nonfunctional tumours. Demographic features are listed in Table 1. One hundred patients had the primary tumour resected, and 48 liver surgeries were performed in 37 patients.  Table 2 lists all the interventions that occurred during the study cohort. Complete histological analysis was available in 76 cases, all of which were either G1 or G2 tumours. The remaining cases were all histologically confirmed neuroendocrine tumours; however, Ki67 analysis was not available in these cases. 

### 3.2. Primary Tumour Resection and Survival

**Figure 1:**
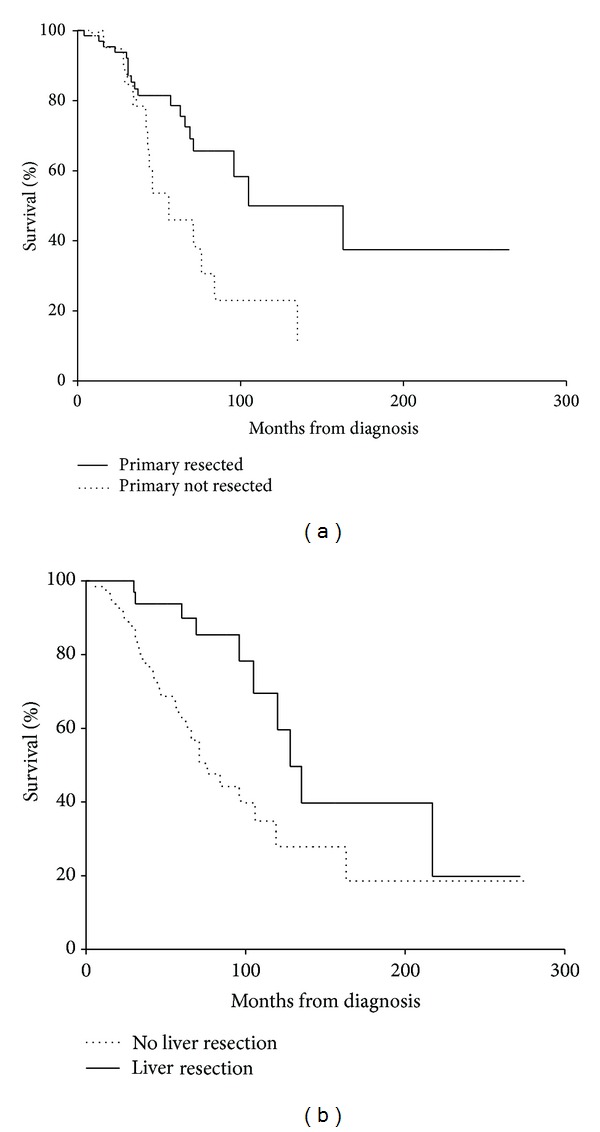
(a) Kaplan-Meier survival curves comparing patients who have primary tumour resection to patients in whom the primary tumor was not resected (120 months versus 56 months, *P* < 0.05). (b) Kaplan-Meier survival curve comparing patients with metastatic small bowel NET who underwent a liver resection to those with hepatic metastases at presentation who did not have liver resection (128 months versus 76 months, *P* < 0.05).

Primary tumour resection was defined as resection of the primary tumour and if surgically possible resection of associated mesenteric mass and lymph node disease. Not all primary resections were performed at the centre; however, histological assessment of the resected specimen was performed at King's College Hospital in these cases. One hundred patients had the primary resected, and 4 patients had attempted resection of the primary but at laparotomy were found to be irresectable; there were no postoperative deaths within 30 days of surgery. Kaplan-Meier curves were constructed to determine if there was any survival benefit for patients in whom the primary tumour was resected (Figure 1(a)). There was improved survival in patients who underwent resection of primary tumour compared to those in whom the primary remained (120 versus 56 months, *P* < 0.05). 

There was survival benefit in resection of the primary tumour in patients with stage 4 disease at the presentation compared to those in whom the primary was not resected (105 versus 56 months, *P* < 0.05). 

Of patients that did not undergo resection of the primary tumour the reasons were as follows: 9 cases were due to the primary being regarded as irresectable; 4 patients had attempted resection, however, at laparotomy the primary could not be removed; and 2 were due to not being considered for primary resection due to comorbidity. In one case the patient declined to surgery opting for conservative management. In the remaining cases surgery was not considered for the primary due to the volume of metastatic disease and/or impaired functional status due to carcinoid heart disease. 

### 3.3. Liver Resection and Survival

Thirty-seven patients had a total of 48 liver resection surgeries, including 2 patients who had a liver transplant.  Table 3 lists the different types of liver surgery that patients underwent. The median age of patients undergoing liver resection was 55 years (range 32–77). All patients that underwent liver resection had the primary tumour resected previously or at the time of liver surgery. Of the 37 patients undergoing liver surgery 14 patients also had the primary removed at the time of surgery. The following complications occurred postsurgery: 1 patient died within 30 days postprocedure. 1 patient had a postoperative-bleed, 1 bile leak, and 1 wound infection which required drainage. Two patients underwent liver transplant during this study: one patient died 5 months following the surgery, due to development of pneumonia, and the other patient remains disease-free at 240 months posttransplant. There was a significant survival benefit in patients who underwent liver resection of hepatic metastases with stage 4 small bowel NETs compared to patients with stage 4 small bowel NETs and hepatic metastases who did not have liver resection (128 versus 76 months, *P* < 0.05), Figure 1(b). 

### 3.4. Prognostic Factors

On univariate analysis, the following clinicopathological features were related to improved prognosis: not having carcinoid heart disease, resection of primary tumour, G1 histological grade, and liver resection. There was no difference in survival for the following factors: gender, uptake on octreoscan, presence of a functional syndrome, and treatment with somatostatin analogues. Multivariate analysis was performed using Cox regression analysis adjusting for all factors that showed a significant difference on univariate analysis. Primary tumour resection and no carcinoid heart disease were identified as the only independent predictors of survival. Not having the primary tumour resected was associated with a relative risk of 2.9 (1.3–6.1), *P* < 0.005. Not having carcinoid heart disease was associated with a decreased relative risk 0.145 (0.06–0.36), *P* < 0.005. 

### 3.5. TNM Staging and Survival

**Figure 2:**
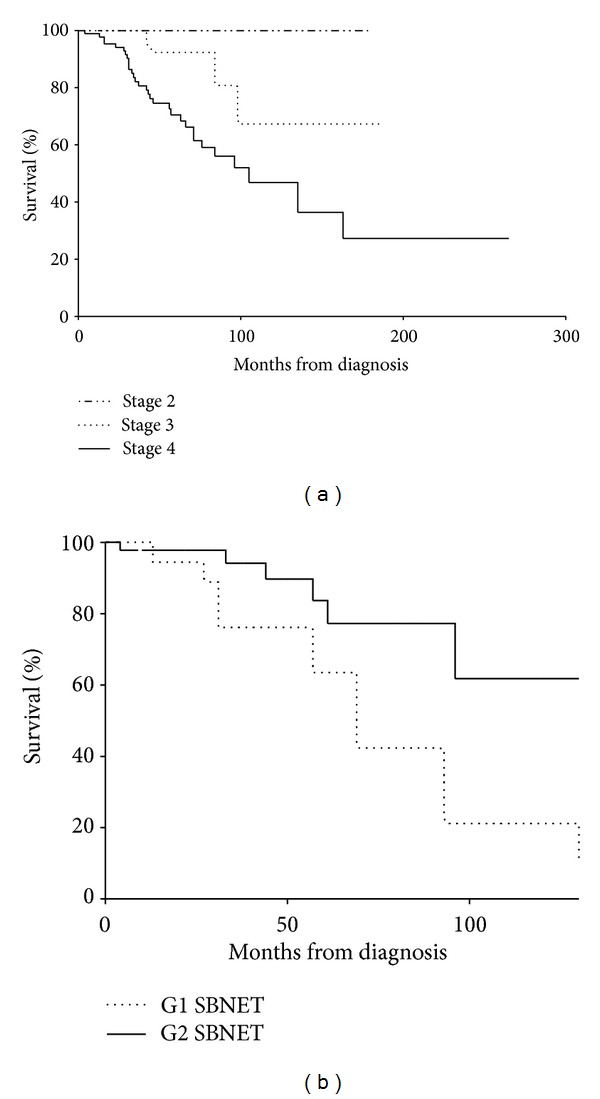
(a) Cumulative small bowel NET survival according to TNM staging. Stage 2 versus stage 3 *P* > 0.05 (nonsignificant), stage 2 + stage 3 versus stage 4 (*P* < 0.05), stage 3 versus stage 4 (*P* < 0.05). (b) Cumulative small bowel NET survival curve assessing histological grade. G1 small bowel NETs had significantly better prognosis than G2 NETs (undefined versus 69 months, *P* < 0.05).

The TNM staging demonstrated significant difference in survival between stage 2 and 3 versus stage 4 disease (*P* < 0.05) and also stage 3 versus stage 4 disease (*P* < 0.05). There was no significance in survival between stage 2 and stage 3 disease (Figure 2(a)). Therefore, improved survival in patients with localised/locoregional disease compared to patients with metastatic disease at the presentation was demonstrated.

There was significantly improved prognosis between G1 compared to G2 tumours, *P* < 0.05. There were no patients with G3 tumours for assessment, see Figure 2(b).

### 3.6. Development of Recurrent Disease

There were 4 patients with TNM stage 2 disease, 23 patients with TNM stage 3 disease, and 10 patients in whom the staging was not known but had no evidence of residual disease postoperatively and were regarded as R0/R1 resection. The remaining patients had clear evidence of distant metastatic disease prior to undergoing resection of the primary tumour. 

Of the patients who underwent attempted curative resection without distal metastatic disease there were 36 patients suitable for analysis. Of these 11 (30.6%) patients have developed recurrent disease. Median period for development of recurrence was 55 months (range 11–122). There was no recurrence in patients with stage 2 disease, and median duration of followup was 37 months (17–180 months). Median followup for patients with stage 3 disease was 37.5 months (range 6–119 months). Recurrence occurred in 7 of 23 (30.4%) patients, and median duration to recurrence was 47 months (range 11–96 months). 

### 3.7. Overall Survival and Cause of Death

The 5-year and 10-year survivals were 79.5% and 48.5%, respectively, for all patients independent of the stage of the disease. The median survival for patients with stage 4 disease was 98 months, with 5-year survival of 74.5%. 

There were 44 (32.8%) deaths during the follow-up period of the study. The cause of death is displayed in Table 4. 20.5% of patients died from nontumour-related deaths; the most common causes were cardiovascular death and a second malignancy. The cause of death could not be identified in one case. 

## 4. Discussion

We have demonstrated that the ENETS TNM staging system for midgut offers prognostic information, with the worst survival demonstrated in patients with stage 4 disease compared with stage 2 or stage 3 disease. Furthermore, the proposed grading system based on Ki67 and mitotic index provided statistically different prognosis between G1 and G2 NETs. 

No difference in survival was demonstrated between stage 2 and stage 3 disease; this could be in part due to the duration of followup and small numbers of cases with stage 2 disease. However, other studies have not demonstrated difference in survival between stage 2 and stage 3 disease [13]. Markers of cellular proliferation as measured by Ki67 index and mitotic rate were incorporated in to the novel ENETS TNM staging and grading system. Studies demonstrated these markers as predictors of survival in pancreatic and upper gastrointestinal NETs and hence their incorporation into the ENETs guidance [11, 22, 23]. In this study we demonstrated significant improvement in survival for patients with G1 compared to those with G2 NETs; this supports the findings in other studies [14, 24].

Overall the 5-year and 10-year survivals were 79.5% and 48.5%, respectively, for all patients independent of stage of disease. This shows improvement in the 5-year survival when compared to previous studies, including the SEER data [6]. There appears to be a trend towards improvement in survival when looking at the 5-year survival data from recently published data looking at patient cohorts over the last two decades [6, 12, 14]. The causes for this improved survival have not been fully elucidated. It may be in part related to increased use of somatostatin analogues and their antiproliferative effect. Secondly, the management of patients in multidisciplinary teams and the more aggressive surgical management of these patients may have improved survival. The study cohort may be biased as it is not a population-based survival but instead a survival of patients managed in a specialist centre. Finally, lead time bias may also be a factor; however, there is no clear evidence to support that patients are being diagnosed at an earlier stage of the disease. 

The cause of death in this study demonstrated that 47.3% were related to tumour progression which is similar to that published in the UKINETs study [14]. A similar percentage of patients died from carcinoid heart disease and small bowel obstruction [14, 25–27]. Interestingly 20.5% of patients died from tumour-unrelated causes, which is similar to that identified from previous studies [14, 28]; in part it could be related to the longer survival of patients with small bowel NETs, leading to other causes of death accounting to a large percentage of deaths. 

This study demonstrates improved survival in patients in whom the primary tumour was resected, with univariate and multivariate analyses. Furthermore, there was improved survival for patients who presented with stage 4 disease who had resection of primary tumour compared to those in whom the primary was not resected. Previous studies have demonstrated a survival benefit in resection of primary tumour; however, the study population has been rather heterogenous [29]. A recent systematic review by Capurso et al. [17] concluded that there is a possible benefit of resection of the primary lesion in patients with unresectable liver metastases. 

This study demonstrates significant prognostic benefit of liver resection on survival using univariate analysis, though this is not a prognostic factor with multivariate analysis. Numerous studies have demonstrated improved symptom control and quality of life following hepatic resection and tumour debulking [22, 30–32]. The role of hepatic surgery in patients with metastatic NETs should still be considered in appropriate candidates [14, 18]. A number of studies have demonstrated improved prognosis following liver resection in patients with small bowel NETs in a univariate analysis [14, 16, 33].

There is limited data regarding the time to develop recurrent disease following “curative resection” in patients with local/locoregional disease. We demonstrated no evidence of recurrence in patients with stage 2 disease following resection. Similar findings have been reported in other studies [24, 34]. Following what was regarded as R0/R1 resection we have demonstrated that median time to development of recurrence is 55 months. Approximately 30% of patients with stage 3 disease will develop recurrence within a median period of 47 months. Studies by Le Roux and Landerholm have demonstrated recurrence rates around 30% following complete resection of primary tumour and locoregional disease [28, 34]. Followup of patients with stage 2 or 3 tumours should be extended beyond 5 years [18]. Duration of followup for patients with stage 1 disease remains unclear. 

This study is a single centre experience that has certain inherent limitations; due to the nature of referrals it was not possible to accurately stage all patients who underwent surgical resection and perform complete histological analysis. The rate of disease recurrence following resection of locoregional disease may be overestimated due to referral bias; however, similar rates have been reported in other studies. 

## 5. Conclusion

This study has demonstrated that ENETS TNM staging and grading system is able to prognosticate between stage of disease and grade of tumour. Primary tumour resection and not having a carcinoid heart disease are both prognostic factors on multivariate analysis. There may potentially be a role of determining the likelihood of recurrence following surgery using the TNM staging system. Finally, overall survival for small bowel NETs seems to be improving.

## Figures and Tables

**Table 1:** Patient and tumour demographics.

Characteristic	Total group	Male	Female	% of all SBNETs
Site of primary tumor				
Duodenum	3	3	0	2.2
Jejunum	4	2	2	3
Ileum	131	63	68	94.8
Total	138	68	70	
TNM stage at diagnosis				
Stage 1	0	0	0	0
Stage 2	4	3	1	3
Stage 3	23	10	13	16.7
Stage 4	91	42	49	66
Stage unknown	20	13	7	14.5
Median age	65	67	64	
Age range	29–87	31–87	29–82	
Tumour grade				
G1	51	22	29	
G2	25	15	10	
G3	0	0	0	
Not available	62	31	31	
Functional tumour	76			55
Nonfunctional tumour	62			45

SBNETs: small bowel NETs.

**Table 2:** List of different interventions undertaken in small bowel neuroendocrine tumour patients; it lists the intervention and number of procedures undertaken.

Intervention	No. of interventions	No. of patients
Total number that had primary tumour resected	100	100
Failed resection of primary tumour	4	4
No resection of primary tumour	34	34
Primary tumour resection plus liver resection/RFA	14	14
Resection of liver metastases	48	37
Resection of other sites of metastatic disease	2	2
Liver transplant	2	2
Carcinoid heart valve surgery	3	3
Further bowel resection	3	3
TACE/TAE	23	17
SIRT	7	6
PRRT	19	16
^ 131^I-MIBG therapy	14	14
Radiotherapy	2	2
Chemotherapy	10	10

For ^131^I-MIBG (iodine-131-meta-iodobenzylguanidine) and PRRT (peptide receptor radiotargetted therapy) an intervention comprised of 3-4 cycles of therapy. Each embolization was counted as a separate intervention. Heart value surgery involved tricuspid valve replacement in 2 cases, and one case had a tricuspid valve replacement plus pulmonary valvuloplasty. Abdominal radiotherapy was performed in two patients.

**Table 3:** List of the types of liver resection undertaken in patients. The 2 stage surgery is counted as two separate individual surgeries.

Liver surgery	Number of surgeries
Right hepatectomy	12
Right hepatectomy ± wedge resection or RFA	9
Left hepatectomy	4
Left hepatectomy ± wedge resection or RFA	4
Nonanatomical resection/metastasectomy/wedge resection	7
Stage 2 Liver surgery (hemihepatectomy plus portal vein ligation)	2
Liver transplant	2
RFA	4
Partial right hepatectomy	1
Partial left hepatectomy	1

RFA: radiofrequency ablation.

**Table 4:** Cause of death in patients with small bowel neuroendocrine tumours.

Cause of death	Number of patients	% of all deaths
Tumour burden	21	47.7
Small bowel obstruction	6	13.6
Intervention related (30 day)	2	4.5
Carcinoid heart disease	5	11.4
Tumour unrelated cause	9	20.5
Unknown	1	2.7

Total	44	100

## References

[1] Hatzaras I, Palesty JA, Abir F (2007). Small-bowel tumors: epidemiologic and clinical characteristics of 1260 cases from the Connecticut tumor registry. *Archives of Surgery*.

[2] Modlin IM, Champaneria MC, Chan AKC, Kidd M (2007). A three-decade analysis of 3,911 small intestinal neuroendocrine tumors: the rapid pace of no progress. *American Journal of Gastroenterology*.

[3] Modlin IM, Oberg K, Chung DC (2008). Gastroenteropancreatic neuroendocrine tumours. *The Lancet Oncology*.

[4] Hauso O, Gustafsson BI, Kidd M (2008). Neuroendocrine tumor epidemiology: contrasting Norway and North America. *Cancer*.

[5] Modlin IM, Lye KD, Kidd M (2003). A 5-decade analysis of 13,715 carcinoid tumors. *Cancer*.

[6] Yao JC, Hassan M, Phan A (2008). One hundred years after “carcinoid”: epidemiology of and prognostic factors for neuroendocrine tumors in 35,825 cases in the United States. *Journal of Clinical Oncology*.

[7] Ramage JK, Davies AHG, Ardill J (2005). Guidelines for the management of gastroenteropancreatic neuroendocrine (including carcinoid) tumours. *Gut*.

[8] Srirajaskanthan R, Shanmugabavan D, Ramage JK (2010). Carcinoid syndrome. *The British Medical Journal*.

[9] Nilsson O, Kölby L, Wängberg B (1998). Comparative studies on the expression of somatostatin receptor subtypes, outcome of octreotide scintigraphy and response to octreotide treatment in patients with carcinoid tumours. *British Journal of Cancer*.

[10] Norheim I, Oberg K, Theodorsson-Norheim E (1987). Malignant carcinoid tumors. An analysis of 103 patients with regard to tumor localization, hormone production, and survival. *Annals of Surgery*.

[11] Rindi G, Klöppel G, Couvelard A (2007). TNM staging of midgut and hindgut (neuro) endocrine tumors: a consensus proposal including a grading system. *Virchows Archiv*.

[12] Bergestuen DS, Aabakken L, Holm K, Vatn M, Thiis-Evensen E (2009). Small intestinal neuroendocrine tumors: prognostic factors and survival. *Scandinavian Journal of Gastroenterology*.

[13] Jann H, Roll S, Couvelard A (2011). Neuroendocrine tumors of midgut and hindgut origin: tumor-node-metastasis classification determines clinical outcome. *Cancer*.

[14] Ahmed A, Turner G, King B (2009). Midgut neuroendocrine tumours with liver metastases: results of the UKINETS study. *Endocrine-Related Cancer*.

[15] Curran T, Tulin-Silver S, Patel K (2011). Prognostic clinicopathologic factors in longitudinally followed patients with metastatic small bowel carcinoid tumors. *Pancreas*.

[16] Strosberg J, Gardner N, Kvols L (2009). Survival and prognostic factor analysis of 146 metastatic neuroendocrine tumors of the mid-gut. *Neuroendocrinology*.

[17] Capurso G, Rinzivillo M, Bettini R, Boninsegna L, Fave GD, Falconi M (2012). Systematic review of resection of primary midgut carcinoid tumour in patients with unresectable liver metastases. *The British Journal of Surgery*.

[18] Ramage JK, Ahmed A, Ardill J (2012). Guidelines for the management of gastroenteropancreatic neuroendocrine (including carcinoid) tumours (NETs). *Gut*.

[19] Pape UF, Jann H, Müller-Nordhorn J (2008). Prognostic relevance of a novel TNM classification system for upper gastroenteropancreatic neuroendocrine tumors. *Cancer*.

[20] Burke AP, Thomas RM, Elsayed AM, Sobin LH (1997). Carcinoids of the jejunum and ileum: an immunohistochemical and clinicopathologic study of 167 cases. *Cancer*.

[21] Rindi G, de Herder WW, O’Toole D, Wiedenmann B (2007). Consensus guidelines for the management of patients with digestive neuroendocrine tumors: why such guidelines and how we went about it. *Neuroendocrinology*.

[22] Amarapurkar AD, Davies A, Ramage JK, Stangou AJ, Wight DGD, Portmann BC (2003). Proliferation of antigen MIB-1 in metastatic carcinoid tumours removed at liver transplantation: relevance to prognosis. *European Journal of Gastroenterology and Hepatology*.

[23] Rindi G, Capella C, Solcia E (2000). Introduction to a revised clinicopathological classification of neuroendocrine tumors of the gastroenteropancreatic tract. *Quarterly Journal of Nuclear Medicine*.

[24] Norlen O, Stalberg P, Oberg K (2012). Long-term results of surgery for small intestinal neuroendocrine tumors at a tertiary referral center. *World Journal of Surgery*.

[25] Bhattacharyya S, Davar J, Dreyfus G, Caplin ME (2007). Carcinoid heart disease. *Circulation*.

[26] Diaco DS, Hajarizadeh H, Mueller CR, Fletcher WS, Pommier RF, Woltering EA (1995). Treatment of metastatic carcinoid tumors using multimodality therapy of octreotide acetate, intra-arterial chemotherapy, and hepatic arterial chemoembolization. *The American Journal of Surgery*.

[27] Durante C, Boukheris H, Dromain C (2009). Prognostic factors influencing survival from metastatic (stage IV) gastroenteropancreatic well-differentiated endocrine carcinoma. *Endocrine-Related Cancer*.

[28] Landerholm K, Zar N, Andersson RE, Falkmer SE, Jrhult J (2011). Survival and prognostic factors in patients with small bowel carcinoid tumour. *British Journal of Surgery*.

[29] Hellman P, Lundström T, Öhrvall U (2002). Effect of surgery on the outcome of midgut carcinoid disease with lymph node and liver metastases. *World Journal of Surgery*.

[30] Osborne DA, Zervos EE, Strosberg J (2006). Improved outcome with cytoreduction versus embolization for symptomatic hepatic metastases of carcinoid and neuroendocrine tumors. *Annals of Surgical Oncology*.

[31] Sarmiento JM, Heywood G, Rubin J, Ilstrup DM, Nagorney DM, Que FG (2003). Surgical treatment of neuroendocrine metastases to the liver: a plea for resection to increase survival. *Journal of the American College of Surgeons*.

[32] Srirajaskanthan R, Toumpanakis C, Meyer T, Caplin ME (2009). Review article: future therapies for management of metastatic gastroenteropancreatic neuroendocrine tumours. *Alimentary Pharmacology and Therapeutics*.

[33] Saxena A, Chua TC, Zhao J, Morris DL (2012). Liver-directed therapy for neuroendocrine neoplasm hepatic metastasis prolongs survival following progression after initial surgery. *Journal of Surgical Oncology*.

[34] Le RC, Lombard-Bohas C, Delmas C (2011). Relapse factors for ileal neuroendocrine tumours after curative surgery: a retrospective French multicentre study. *Digestive and Liver Disease*.

